# Targeted metabolomics and medication classification data from participants in the ADNI1 cohort

**DOI:** 10.1038/sdata.2017.140

**Published:** 2017-10-17

**Authors:** Lisa St John-Williams, Colette Blach, Jon B. Toledo, Daniel M. Rotroff, Sungeun Kim, Kristaps Klavins, Rebecca Baillie, Xianlin Han, Siamak Mahmoudiandehkordi, John Jack, Tyler J. Massaro, Joseph E. Lucas, Gregory Louie, Alison A. Motsinger-Reif, Shannon L. Risacher, Alzheimer’s Disease Neuroimaging Initiative, Alzheimer’s Disease Metabolomics Consortium, Andrew J. Saykin, Gabi Kastenmüller, Matthias Arnold, Therese Koal, M. Arthur Moseley, Lara M. Mangravite, Mette A. Peters, Jessica D. Tenenbaum, J. Will Thompson, Rima Kaddurah-Daouk

**Affiliations:** 1 Proteomics and Metabolomics Shared Resource, Center for Genomic and Computational Biology, Duke University, Durham, NC 27710, USA; 2 Duke Molecular Physiology Institute, Duke University, Durham, NC 27701, USA; 3 Department of Pathology and Laboratory Medicine, University of Pennsylvania, Philadelphia, PA 19104, USA; 4 Department of Neurology, Houston Methodist Hospital, Houston, TX 77030, USA; 5 Bioinformatics Research Center, Department of Statistics, North Carolina State University, Raleigh, NC 27607, USA; 6 Department of Radiology and Imaging Sciences and the Indiana Alzheimer Disease Center, Indiana University School of Medicine, Indianapolis, IN 46202, USA; 7 Department of Electrical and Computer Engineering, State University of New York, Oswego, NY 13126, USA; 8 BIOCRATES Life Sciences AG, Innsbruck 6020, Austria; 9 Rosa and Co LLC, San Carlos, CA 94070, USA; 10 Sanford Burnham Prebys Medical Discovery Institute, Orlando, FL 32827, USA; 11 Department of Psychiatry and Behavioral Sciences, and the Duke Institute for Brain Sciences, Duke University, Durham, NC 27710, USA; 12 Duke Social Sciences Research Institute, Duke University, Durham, NC 27708, USA; 13 Institute of Bioinformatics and Systems Biology, Helmholtz Zentrum München, German Research Center for Environmental Health, Neuherberg D-85764, Germany; 14 German Center for Diabetes Research, Neuherberg D-85764, Germany; 15 Sage Bionetworks, Seattle, WA 98109, USA; 16 Department of Biostatistics and Bioinformatics, Duke University, Durham, NC 27710, USA

**Keywords:** Metabolomics, Data publication and archiving, Alzheimer's disease, Lipidomics

## Abstract

Alzheimer’s disease (AD) is the most common neurodegenerative disease presenting major health and economic challenges that continue to grow. Mechanisms of disease are poorly understood but significant data point to metabolic defects that might contribute to disease pathogenesis. The Alzheimer Disease Metabolomics Consortium (ADMC) in partnership with Alzheimer Disease Neuroimaging Initiative (ADNI) is creating a comprehensive biochemical database for AD. Using targeted and non- targeted metabolomics and lipidomics platforms we are mapping metabolic pathway and network failures across the trajectory of disease. In this report we present quantitative metabolomics data generated on serum from 199 control, 356 mild cognitive impairment and 175 AD subjects enrolled in ADNI1 using AbsoluteIDQ-p180 platform, along with the pipeline for data preprocessing and medication classification for confound correction. The dataset presented here is the first of eight metabolomics datasets being generated for broad biochemical investigation of the AD metabolome. We expect that these collective metabolomics datasets will provide valuable resources for researchers to identify novel molecular mechanisms contributing to AD pathogenesis and disease phenotypes.

## Background & Summary

Alzheimer’s disease is a degenerative brain disorder and the most common cause of dementia, presenting as the most common neurodegenerative disease in the United States^1,2^. It is characterized by a decline in memory, language, problem-solving and other cognitive skills that affects a person’s ability to perform everyday activities^3^. Data suggests that pathophysiological changes associated with AD begin decades before the emergence of clinical symptoms^4,5^. AD is becoming an increasing health burden in the United States and globally due to population aging^6^.

The disease is defined by the presence of tau neurofibrillary tangles and Aβ plaques, but coincident pathologies like Lewy body disease, vascular pathology and TDP-43 (transactive response DNA-binding protein 43) deposits are commonly found in AD patients. Current symptomatic therapeutic treatments have modest effects and do not modify the disease course. Researchers hope to develop therapies targeting specific genetic, molecular, and cellular mechanisms so that the actual underlying cause of the disease can be slowed or prevented but currently our understanding of disease mechanisms remains limited. While the majority of AD clinical trials to date have focused on Aβ treatments, other therapeutic approaches are necessary. Understanding biochemical trajectory of disease and metabolic changes related to Aβ and Tau pathology and cognitive decline is essential to advance our understanding of AD etiology as well as for developing novel approaches for drug development.

Recent advances in analytical chemistry led to the emergence of a new field called metabolomics. Metabolomics allows simultaneous measurement of 100’s to 1,000’s of metabolites for mapping perturbations in interconnected pathways and in metabolic networks enabling a systems approach to the study of AD^7,8^. An emerging body of evidence data supports the potential of metabolomics to provide added information for the prediction of AD and has identified a number of potentially important biochemical pathways in AD^9–15^. Though promising, in many cases to this point cohorts are quite small with little replication, and therefore do not allow the effects of confounds such as medications to be addressed. Larger and more comprehensive cohorts are needed to provide the statistical power to detect and develop robust predictive metabolomics models, while also accounting for confounding effects related to medication intake, gender and aging. The Alzheimer’s Disease Neuroimaging Initiative (ADNI) unites researchers around the globe to define the progression of Alzheimer’s disease. ADNI researchers collect, validate and utilize data such as MRI and PET images, genetics, cognitive tests, CSF and blood biomarkers from thousands of subjects as predictors for the disease^16,17^. The Alzheimer’s Disease Metabolomics Consortium (ADMC) has the goal of building a comprehensive metabolomics database for AD in partnership with ADNI. This will be a national resource for the Alzheimer’s community that enables interrogation of global metabolic changes within a pathway and network context and where metabolomics data can be used to compliment and inform genomics and imaging data. Eight targeted and non-targeted metabolomics platforms that have their own strengths and limitations are being used to profile thousands of ADNI subjects.

For this dataset, we used a targeted, widely utilized and cross-validated metabolomics platform AbsoluteIDQ^®^-p180 (Biocrates AG) to profile baseline serum samples from ADNI1 cohort where vast data exist on each patient including cognitive decline and imaging changes over many years, information on CSF markers, genetics and other omics data. The goal of this data generation is to aid in the discovery of metabolic failures correlated with disease and progression and biomarkers for a range of important physiological processes in AD. We describe a foundation for automated curation of metabolomics data, including R scripts for removing analytes with poor precision or with significant missing values, and samples that have missing clinical data or are metabolic outliers. We address approaches within this cohort for dealing with confounds that impact metabolomics data and findings including medications, broadly applicable to pharmacometabolomic investigations^18–20^.

## Methods

### Alzheimer’s disease neuroimaging initiative (ADNI) cohort

Clinical and demographic data used for this study were obtained through the ADNI data repository (http://adni.loni.ucla.edu/). Written informed consent was obtained for all participants and prior Institutional Review Board approval was obtained at each participating institution. All demographic information, neuropsychological and clinical assessment data, and diagnostic information used in this study are available from the ADNI clinical data repository (http://adni.loni.ucla.edu/). Information about ADNI can be found in Petersen *et al.*^16^ and at http://www.adni-info.org/^16^. Key clinical and demographic variables for the ADNI1 participants as of May 2016 are summarized in Table 1 and available through Synapse (see Table 2). Note that ADNI data collection is ongoing, so variables on LONI may have been updated since that subset was downloaded. We have included this snapshot in order to enable analytic reproducibility despite a dynamic source of truth.

### Serum collection and sample management

Morning fasting blood samples from the baseline visit were included in the study (all but 69 were fasting). Samples were collected in two bar-coded 10 ml red-top plastic Vacutainer blood tubes, blood was allowed to clot for 30 min followed by a 15 min centrifugation at 3,000 rpm (1,500 rcf) as described in the ADNI standard operating procedures (www.adni-info.org). Then the serum was transferred into a bar-coded 13 ml polypropylene transfer tube and capped and allowed to freeze in dry ice. Samples were shipped overnight to the ADNI biomarker core laboratory at the University of Pennsylvania Medical Center. Samples were thawed once in the core facility and aliquoted to 0.5 ml samples, then subsequently aliquoted once more for individual laboratory analyses. A 20 μl sample aliquot was delivered to the Duke Proteomics and Metabolomics Shared Resource for analysis with the p180 platform.

### Metabolomics analysis and QC using the AbsoluteIDQ p180 kit

#### Sample preparation

**Figure 1:**
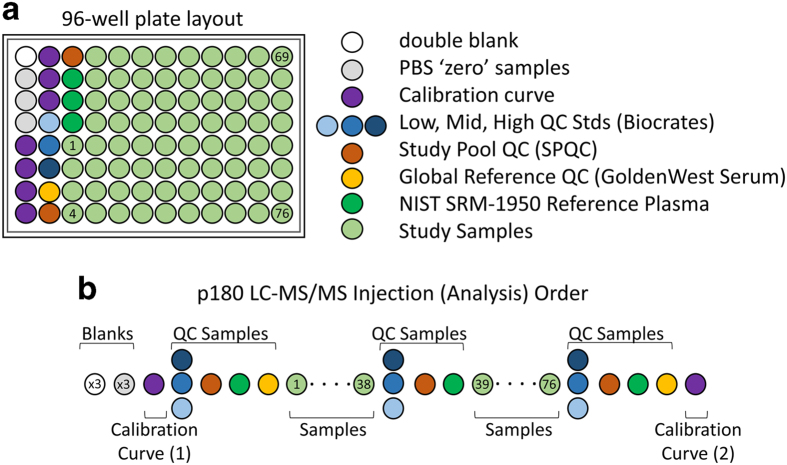
Plate layout for participant and quality control samples. (**a**) 96-well plate layout used for sample preparation and data collection for the Absolute IDQ p180 metabolomics analysis. Each of the eleven plates (*n*=833 study samples) analyzed in the study used the same lot of calibrators, Biocrates QCs, study pool QC (SPQC), GoldenWest Serum and NIST SRM-1950 plasma. (**b**) Analysis order for each plate, showing how the calibration curve and QC samples bracket the actual sample analyses in order to decrease the likelihood of intraplate bias. LC-MS/MS and FIA-MS/MS use the same analysis order, but FIA-MS/MS excludes the calibration curve.

Samples were prepared and analyzed in the Duke Proteomics and Metabolomics Shared Resource using the Absolute*IDQ* p180 kit (Biocrates Life Sciences AG, Innsbruck, Austria) in accordance with the user manual. In brief, after the addition of 10 μl of the supplied internal standard solution to each well on a filterspot of the 96-well extraction plate, 10 μl of each serum sample, quality control (QC) samples, blank, zero sample, or calibration standard were added to the appropriate wells (Fig. 1). The plate was then dried under a gentle stream of nitrogen. The samples were derivatized with phenyl isothiocyanate (PITC) for the amino acids and biogenic amines, and dried again. Sample extract elution was performed with 5 mM ammonium acetate in methanol. Sample extracts were diluted with either 40% methanol in water for the UPLC-MS/MS analysis (15:1) or kit running solvent (Biocrates Life Sciences AG) for flow injection analysis (FIA)-MS/MS (20:1).

#### Quality control samples

The analysis of the samples using the AbsoluteIDQ p180 kit was performed using four specific sets of quality controls. First, low/mid/high level QC samples provided by Biocrates Life Sciences AG were prepared and analyzed on each plate as recommended by the manufacturer. These QC samples were used for a technical validation of each kit plate. Second, to allow appropriate inter-plate abundance scaling based specifically on this cohort of samples, we generated a Study Pool QC (SPQC) by combining approximately 10 μl from the first 76 samples for analysis. This sample was frozen in aliquots of 25 ul then prepared and analyzed twice on each plate. Third, there were 20 blinded analytical duplicates obtained from the same serum draw scattered throughout the study in a manner blinded to the investigators until data was sent to the ADNI informatics core for unblinding. The commonly used reference materials NIST SRM-1950 plasma (*n*=3 per plate) and GoldenWest serum pool (*n*=1 per plate) were also analyzed on each plate to allow cross-comparison against other sample cohorts in the future.

Figure 1a shows the preparation layout for the 96-well plates as utilized in this study. In total, eleven plates were prepared in order to analyze 831 serum samples. The blank, zero sample, calibration standards, and Low/Mid/High QC samples provided with the kit were arranged as recommended by Biocrates. In order to improve the ability to compare results with other metabolomics studies and reduce plate-to-plate batch effects, six additional wells were used for the additional QC samples as described above: two wells for the study pool QC (SPQC), one well for the GoldenWest Pooled Serum Standard^21^, and three wells for the NIST SRM-1950 Standard Reference Plasma. The remaining 76 wells were used for cohort samples. The analysis order of each plate is summarized in Fig. 1b, and was arranged to maximize quantitative accuracy and precision within a plate and limit the potential for batch effects. The analysis order included running the standard curve twice, once at the beginning and end of the samples (LC-MS/MS only). For both LC-MS/MS and FIA-MS/MS analysis, the Biocrates QC’s and Goldenwest Serum QC were prepared once but injected in technical triplicate, once before, in the middle (after 38 samples) and at the end of the sample set. The SPQC samples (*n*=2) were each analyzed once, with one analysis before and one after all samples on the plate. The NIST SRM-1950 plasma (*n*=3) were also analyzed once each at the beginning, middle, and end of the cohort samples. Bracketing the standard curves and nesting the analytical samples between the QCs offers the best chance of observing any system drift and assuring optimal instrument performance across the sample set.

#### Quantitative UPLC-MS/MS and FIA-MS/MS analysis

Mass spectrometry analysis was performed based on Standard Operating Procedures (SOP #8114) provided by Biocrates for the AbsoluteIDQ p180 kit. Chromatographic separation of amino acids and biogenic amines was performed using an ACQUITY UPLC System (Waters Corporation) using an ACQUITY 2.1 mm×50 mm 1.7 μm BEH C18 column fitted with an ACQUITY BEH C18 1.7 μm VanGuard guard column, and quantified by calibration curve plotting ratio of analyte to internal standard versus standard concentration, fitted using a linear regression with 1/x weighting. All amino acids and biogenic amines utilize either deuterated or ^13^C stable-isotope labeled internal standard of the exact analyte or closely-eluting compound of similar class. Acylcarnitines, sphingolipids, and glycerophospholipids were analyzed by flow injection analysis tandem mass spectrometry (FIA-MS/MS) and quantified by internal standard calibration; eight separate internal standards are used to quantify the various acylcarnitines, while a single internal standard is used for each of the other lipid classes. Thus, FIA-MS/MS analytes are reported as semi-quantitative values except where a stable-isotope labeled internal standard of that exact analyte was used. Samples for both UPLC and FIA analyzed using a Xevo TQ-S mass spectrometer (Waters Corporation) using positive electrospray ionization operating in the Multiple Reaction Monitoring (MRM) mode. MRM transitions (compound-specific precursor to product ion transitions) for each analyte and internal standard were collected over a scheduled retention time window using tune files and acquisition methods provided in the AbsoluteIDQ p180 kit. The UPLC data were imported into TargetLynx (Waters Corporation) for peak integration, calibration and concentration calculations. The UPLC data from TargetLynx and FIA data were analyzed using Biocrates’ Met*IDQ* v5.4.8 software. The kit data are reported in detail in the Supplementary Information on LONI, along with a color-coded key denoting samples that were below the limit of detection (<LOD), below the lowest calibration standard (<LLOQ), or quantified based on a ratio to a class-based internal standard (semi-quantitative). The data generated for the study samples, SPQC samples, as well as NIST-SRM 1950 from each plate can be downloaded at http://adni.loni.usc.edu/data-samples/access-data/ (See below).

#### Treatment of isobaric lipids

The p180 kit quantifies individual lipid species the ‘sum lipid composition’ level; that is, the quantity of the lipid reported as PC (36:3) (for example) is the sum of all possible phosphatidylcholine lipids in the sample which have a total of 36 carbons and 3 double bonds. Additionally, analyzed glycerophospholipids are differentiated according to the presence of ester and ether bonds in the glycerol moiety. Typically, the annotation ‘aa’ in lipid nomenclature indicates that both fatty acids at the sn-1 and sn-2 position are bound to the glycerol backbone via ester bonds, whereas ‘ae’ donates that fatty acid in the sn-1 or sn-2 position is bound via an ether bond. Total number of carbon atoms and double bonds present in lipid fatty acid chains are denoted as ‘C x:y’ where x indicates the number of carbons and y the number of double bonds. For the specific glycerophospholipid class sphingomyelins (SM), only the fatty acid bound to glycerol backbone at the sn-2 position are indicated under the standard assumption that sphingosine (d18:1) is bound at the sn-1 position. The FIA-MS/MS analysis with the Biocrates p180 kit is performed using mass spectrometry precursor/product transitions (‘MRMs’) with lipid species-specific precursor ions and class-specific fragment ions, to report quantitative values for a number of lipids at the sum lipid composition level.

Due to the relatively low mass resolution of triple quadrupole MS instruments, the detected flow-injection based MRM signal is a sum of several isobaric lipids within the same class. For example, according to the LIPID MAPS database (www.lipidmaps.org), the signal of PC aa C36:6 can arise from at least 15 different lipid species that have different fatty acid composition (e.g., PC 16:1/20:5 versus PC 18:4/18:2), various positioning of fatty acids sn-1 and sn-2 position (e.g., PC 18:4/18:2 versus PC 18:2/18:4) and different double bond positions in those fatty acid chains (e.g., PC(18:4(6Z,9Z,12Z,15Z)/18:2(9Z,12Z)) versus PC(18:4(9E,11E,13E,15E)/18:2(9Z,12Z))). The Biocrates MetIDQ software applies an isotopologue correction for the obtained lipid data in order to increase accuracy, but due to limitations of FIA-MS/MS data on triple quadrupoles, this correction is incomplete for some lipids^22^. To assist with clarity in the interpretation of the results reported herein using the p180 kit, we have included a list of measured lipids. Their common names and possible isobars and isomers are summarized in Supplementary Table 1: The list of lipids measured with the Absolute*IDQ* p180 kit. Within this table, possible isobars are given within ±0.5 Da range due to the typical conditions under which triple quadrupole mass spectrometers are operated for the kit, and the isobars are reported as sum compositions^23^. The position of acyl chains (sn-1, sn-2) and double bonds are not indicated for the possible isomers. The possible isotopologue interferences are indicated in cursive. For each isomer, examples of LIPID MAPS Structure Database (http://www.lipidmaps.org/data/databases.html) entries are listed, where applicable. The table comprises examples of possible isobars and isomers to the best of our current knowledge, but does not prioritize the subspecies based on likelihood of existence or relative abundance in any particular biological system.

### Data processing

**Figure 2:**
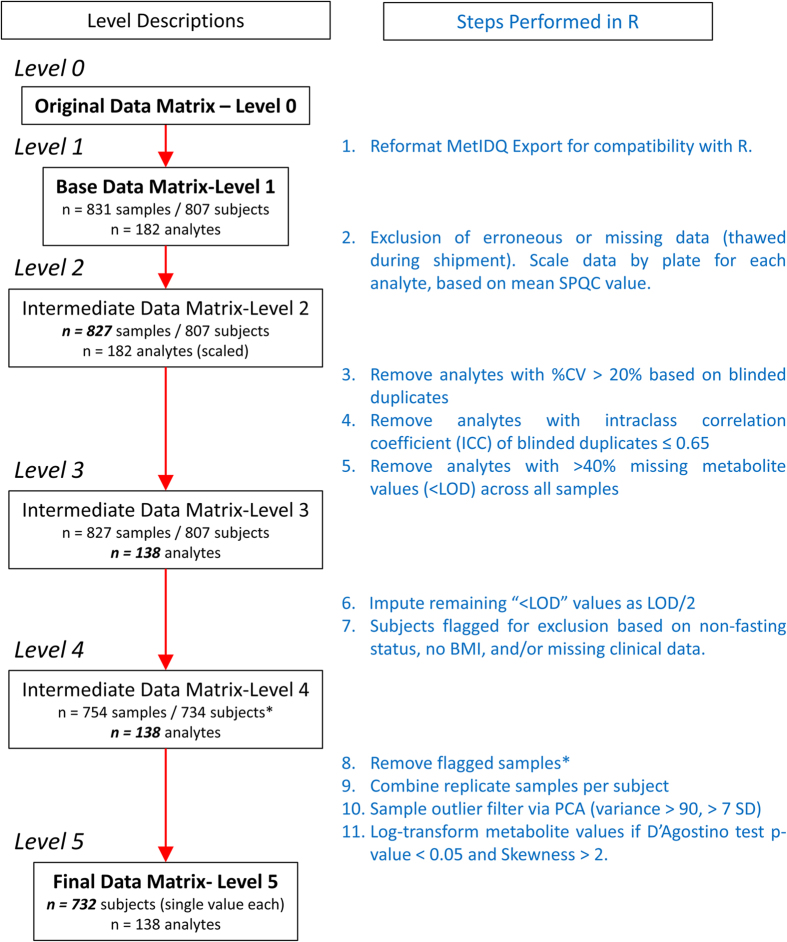
Workflow description for data curation and scaling of the p180 metabolomics analysis of the ADNI1 cohort. The use of Levels (shown at left) breaks the workflow into discrete steps which can be applied to multiple metabolomics data types. The workflow executed in R is described on the right. *Subjects flagged for exclusion in Level 4 are not physically excluded from the table until Level 5.

Statistical preprocessing was performed using the open-source, statistical software, R v3.2.4 (www.r-project.org), with scripts available for download at http://dx.doi.org/10.7303/syn7354353. The processing included the steps briefly described herein, and graphically depicted as a flowchart in Fig. 2.

In the first step we excluded four samples due to erroneous inclusion in the cohort (thawed during shipment), and the values of each analyte were scaled across the different plates using the Study Pool QC (SPQC) duplicates analyzed twice on each plate. Given SPQC duplicates, the correction factor for each analyte in a specific plate was obtained by dividing its global average by its average within the plate to adjust for the batch effects, yielding the Intermediate Data Level 2.

The second set of steps include filtering of analytes based on quality metrics. We applied two criteria to filter individual metabolites as part of our data quality control (QC) evaluation process based on the 20 blinded ADNI duplicates: 1) a coefficient of variation (CV) <20% across plates, and 2) an intraclass correlation coefficient (ICC) >0.65. ICC compared the two measurements for each of the blinded duplicates. Additionally, analytes with >40% of measurements below the lower limit of detection (<LOD) were excluded from the analysis. Combined, these three steps allow only the most robust analytes from the panel through to the Level 3 data matrix, and reduced the total number of analytes reported in the dataset from 182 analytes (Intermediate Data—Level 2) to 138 analytes (Intermediate Data—Level 3). The QC Results for all analytes including LOD percentage, CV of the blinded replicates, and ICC values are reported in the Supplementary Table 2 (FIA-MS/MS) and 3 (UPLC-MS/MS).

The steps between Level 3 and Level 4 in the pipeline (Fig. 2) perform missing value replacement and allow for exclusion of samples due to other missing data that may vary from study to study. Remaining samples with values reported as ‘<LOD’ were imputed using LOD/2 value for each specific analyte. Also, there were 73 samples determined to be pre-analytical outliers for one or more of the following reasons which were flagged and removed from the dataset. These included a total of 69 samples identified as non-fasting, 2 samples lacking corresponding body mass index (BMI) values, and 1 for which no baseline medication record was reported. After these steps, the Intermediate Data—Level 4 contained *n*=754 samples (734 subjects) and *n*=138 analytes.

The last steps in the statistical pretreatment pipeline serve to combine replicate measurements to give one value per biological sample, filter out any statistical outlier subjects, and perform log-transformation if necessary. To obtain a single value for the 20 subjects with blinded duplicates we calculated the average of the duplicates for each subject and reported this single value. We checked for the presence of outlier subjects by performing principal components analysis, and evaluating the subject distance from the centroid in the K-dimensional space based on principal components that explained >90% cumulative variance. Subjects located more than 7 s.d. from the mean were flagged as outliers. This procedure identified two additional samples that were excluded from the final data matrix. Finally, log2 transformation was performed for those analytes which show *P*-value for D’Agostino <0.05 and Skewness test >2. The final preprocessed data matrix (Final Data Matrix—Fig. 2, Level 5) contained data for 732 subjects and 138 analytes.

It should be noted that the statistical curation process described above is very stringent, leaving behind only the most robust analytes but in the process potentially excluding some good measurements. One weakness in our pipeline is that by filtering on intraclass correlation coefficient (ICC) it is possible that we filtered out some robust measurements which simply had very narrow biological measurement range over the blinded samples, precluding the observation of a correlational trend. Examples of this potentially include histamine (17.2% missing values, 5.4% CV, 0.09 ICC) and methionine (0% missing values, 8.1% CV, 0.65 ICC), which almost certainly would have been left in the dataset using many commonly-used and less stringent filtering criteria. To address this shortcoming, future studies are being designed with at least three measurements of each blinded replicate sample and triplicate preps of the SPQC on each plate instead of the NIST SRM-1950, to allow more robust filtering based on imprecision across a wider dynamic range. Metabolites excluded from the analysis suggested here may also be recovered when reanalyzing data in the context of comparative data from other cohorts.

### Collection and curation of medication data

**Figure 3:**
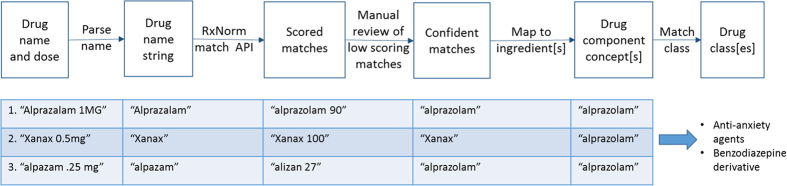
Overview of drug mapping from free text data to medication classes. Drug names are parsed and passed to the RxNorm API to determine approximate string matches. Low scoring matches are reviewed manually. Once the drug, whether brand name or generic, has been mapped to an RxNorm ingredient, corresponding classes are ascertained.

Many classes of medications have been shown to affect metabolism and change levels of certain metabolites^18–20,24^. It is thus necessary to take drug information into account as a potential confounder for metabolomics analysis. In order to convert free text medication information into computable drug data, we applied a pipeline described previously^25^. In brief, as shown in Fig. 3, we employed the National Library of Medicine’s (NLM) RxNorm API (application programming interface) to match drug names extracted from patient medication information containing lexical variations and misspellings to standardized drug concept identifiers. Corresponding concept identifiers are returned along with confidence scores. Low scoring terms were reviewed manually and adjusted as appropriate.

We mapped all versions of a drug, whether brand name or generic, to its respective ingredients, then mapped those ingredients to corresponding drug classes. A subset of drug categories were selected from 3 standardized drug classification systems (NDF-RT, ATC, and MeSH) based on input from experts in Alzheimer’s disease and metabolomics (see Table 3 (available online only)). Criteria for selection of classes to be included in analysis were classes of drugs known to impact metabolomics pathways and/or those likely to be taken by a cognitively impaired population. In this way, each patient was assigned a Boolean flag for whether or not he or she was taking any drug in each respective class. Binary variables can then be used to address potential confounding in subsequent association analyses. The code for this pipeline, including R scripts and API configuration files, is available in Synapse: http://dx.doi.org/10.7303/syn7477310. The final table showing which ADNI1 participants were taking which classes of drugs at their first visit is available at http://dx.doi.org/10.7303/syn7440367.1.

#### Important note

The method described here is the first in a series of iterative approaches to tackle the complex challenge of medications as confounding variables in metabolomic profiling. Medication terminology and software tools continue to evolve. We recommend that those performing future analyses related to medication effects revisit this site and the ADNI data repository for updated curation of medication data.

## Data Records

The primary access site for this dataset is through Sage Bionetworks’ Synapse platform (Data Citation 1). ADNI’s data use agreement prohibits redistribution of ADNI data outside of LONI, so actual data files are hosted by the University of Southern California’s Laboratory of Neuroimaging (LONI). The scripts used for data processing and medication mapping, however, reside in the Synapse platform. Core data files along with associated metadata files, scripts, and Supplementary Files are listed in Table 2. Note that ADNI requires registration to access the data. Researchers may apply for data access at https://ida.loni.usc.edu/collaboration/access/appLicense.jsp.

R Scripts for data processing can be found at http://dx.doi.org/10.7303/syn7354353. Input files (Fig. 2, Level 0) are found in (Data Citation 2) and (Data Citation 3) for FIA and UPLC respectively. Processed data (Fig. 2, Level 5) are available at (Data Citation 4) and (Data Citation 5) for FIA and UPLC respectively. The latest version of clinical annotation data is in (Data Citation 6).

Also included in Supplementary Information on LONI (http://adni.loni.usc.edu/data-samples/access-data/) are the original Excel format exports from the MetIDQ software (Biocrates, Inc). These files include information on calibration ranges, limits of quantification and detection for the assays, and QC sample measurements. They use the original blinded identifiers.

Similarly, the code for the medication mapping pipeline and a link to the current medications file on LONI is available through (Data Citation 7). The medications file must be downloaded and placed in the same directory as the medication mapping R scripts in order to reproduce the medication mapping workflow. The output table showing which patients were taking which classes of drugs is available at (Data Citation 8). Note that, as described in the readme file that accompanies these scripts, manual intervention was used at three points in the pipeline: running RxMix using two different configuration files, and expert review of medication name mapping to accept, reject, or correct results for low-scoring matches.

## Technical Validation

AbsoluteIDQ^®^ p180 kit has been fully validated according to European Medicine Agency Guideline on bioanalytical method validation, and this kit has been utilized in over 200 peer-reviewed publications including a number in dementia and AD^9,13,26^. A recent ring trial showed that inter-laboratory precision was <20% for 82% of the analytes measured with the kit, and 83% of the analytes were accurate within <20%^27^. Additionally, each analyzed kit plate includes an automated technical validation to approve the validity of the run and to provide verification of the actual performance of the applied quantitative procedure including instrumental analysis. Interplate technical validation of each analyzed kit plate was performed using *MetIDQ* software based on results obtained and defined acceptance criteria for blank, zero samples, calibration standards and curves, low/medium/high level QC samples and measured signal intensity of internal standards over the plate. Technical validation for the Xevo TQS was performed according to the following criteria. For the Blank samples the signal intensity for all metabolites and internal standards had to be smaller than a defined minimum value. Signal intensities obtained for zero sample were used for the calculation of plate specific limit of detection (LOD) for FIA-MS/MS analysis. LOD value was defined as concentration that corresponded to three times level of the blank sample. A specific standard measurement (calibrators) was considered valid when calculated concentration was within +/− 30% range of the target concentration. For a specific analyte a minimum of 75% of all calibration standards had to be valid. Biocrates-provided QC samples were human plasma pool spiked with analytes at known concentrations at three different levels. The valid measured concentration range was set for each analyte separately and was within +/− 45% of a target concentration. For a specific analyte a minimum of 67% of all QCs had to be valid as well as a minimum of 50% of all QCs of a certain level had to be valid. Additionally signal intensity for internal standards had to be within valid minimum and maximum intensity value defined by kit manufacturer. All measured plates fulfilled the above described criteria hence confirming the quality and accuracy of the obtained quantitative metabolomics data according to manufacturer recommendations.

To ensure the quality and reproducibility of the quality control and analysis performed prior to data release, independent analysts completed the computational workflow, and achieved reproducibility out to three significant digits across all calculations.

## Usage Notes

Details on how to apply for data access and usage rules can be found at the ADNI website: http://adni.loni.usc.edu/data-samples/access-data/. In brief, users agree to keep the data secure and not to attempt to re-identify research participants. Users also agree to acknowledge ADNI and the ADMC in any derivative publications as follows:

1. On the by-line of the manuscript, after the named authors, include the phrase ‘for the Alzheimer’s Disease Neuroimaging Initiative*’ with the asterisk referring to the following statement and list of names: *Data used in preparation of this article were obtained from the Alzheimer’s Disease Neuroimaging Initiative (ADNI) database (adni.loni.usc.edu). As such, the investigators within the ADNI contributed to the design and implementation of ADNI and/or provided data but did not participate in analysis or writing of this report. A complete listing of ADNI investigators can be found at: http://adni.loni.usc.edu/wp-content/uploads/how_to_apply/ADNI_Acknowledgement_List.pdf
2. On the by-line of the manuscript, after the named authors, include the phrase ‘for the Alzheimer’s Disease Metabolomics Consortium**’ with the double asterisk referring to the following statement and list of names: **Data used in preparation of this article were generated by the Alzheimer’s Disease Metabolomics Consortium (ADMC). As such, the investigators within the ADMC provided data but did not participate in analysis or writing of this report. A complete listing of ADMC investigators can be found at: https://sites.duke.edu/adnimetab/admc-team-directory/
3. The results published here are in whole or in part based on data obtained from the AMPAD Knowledge POrtal accessed at doi:10.7303/syn2580853

Access to scripts and other files described herein that are available through the https://www.synapse.org/#!Synapse:syn2580853/wiki/409840 AMPAD Knowledge Portal hosted on the Sage Bionetworks Synapse informatics data sharing platform, requires adherence to the terms of use described at http://docs.synapse.org/articles/governance.html. Users are required to sign an oath (http://docs.synapse.org/assets/other/oath.html) stating they will not re-identify participants, redistribute the data, or use for advertising and that they will keep data secure, protect privacy, support open access, report any breaches, credit participants, and follow privacy laws.

Because they are managed by different entities, users must register for separate user accounts for LONI (where data are stored) and https://www.synapse.org/#!Synapse:syn2580853/wiki/409840 AMPAD Knowledge Portal on Synapse (repository for scripts and additional information) respectively.

## Consortia

*The Alzheimer Disease Neuroimaging Initiative*

A full list of members is available at http://adni.loni.usc.edu/wp-content/uploads/how_to_apply/ADNI_Acknowledgement_List.pdf.

*The Alzheimer Disease Metabolomics Consortium*

A list of members is available at: https://sites.duke.edu/adnimetab/files/2017/06/ADMCTeamMembersJun14.pdf.

## Additional information

**How to cite this article:** St John-Williams, L. *et al.* Targeted metabolomics and medication classification data from participants in the ADNI1 cohort. *Sci. Data* 4:170140 doi: 10.1038/sdata.2017.140 (2017).

**Publisher’s note:** Springer Nature remains neutral with regard to jurisdictional claims in published maps and institutional affiliations.

## Supplementary Material



Supplementary Table 1

Supplementary Table 2

## Figures and Tables

**Table 1:** Demographics and clinical data of studied ADNI subjects, as determined at baseline.

	**CN (*****n*****=229)**	**MCI (*****n*****=397)**	**AD (*****n*****=193)**	***P*****-value**
Age (years)	75.5 (72.2–78.4)	75.1 (70.1–80.4)	75.8 (70.8–80.5)	0.29
Gender (% male)	47.8%	35.4%	47.6%	0.0019
APOE ε4 (%)	26.5%	53.3%	66.1%	<0.0001
MMSE	29.0 (29.0–30.0)	27.0 (26.0–28.0)	23.0 (22.0–25.0)	<0.0001
ADAS-Cog13	9.33 (6.0–12.3)	18.3 (14.7–23.0)	28.5 (23.7–34.0)	<0.0001
AD, Alzheimer’s disease; ADAS-Cog 13, Alzheimer's Disease Assessment Scale cognitive scale, 13-item version; APOE ε4, Apolipoprotein E; CN. cognitive normal; MCI, mild cognitive impairment; MMSE, Mini-Mental State Examination. *P*-values are based on Chi-square test for APOE status and Kruskal-Wallace for all other variables. *P*-values were not corrected for multiple testing.				

**Table 2:** Names, types, descriptions, and locations of primary data and additional files included in this dataset.

**File Name**	**Description**	**Type**	**Location**	**URL**
Top Level Project Page	Synapse Portal page for AMP-AD ADNI project	Portal	AMPAD Knowledge Portal / Synapse	https://www.synapse.org/#!Synapse:syn5592519
*Primary Metabolomics Files*				
ADMC Duke Biocrates P180 Kit Flow injection analysis	FIA ‘Level 0’ data	Data	LONI	http://dx.doi.org/10.7303/syn7440354.1
ADMC Duke Biocrates P180 Kit Flow injection analysis Dictionary	Data dictionary for p180 FIA	Data Dict	LONI	http://dx.doi.org/10.7303/syn7477315.1
ADMC Duke Biocrates P180 Kit Ultra Performance Liquid Chromatography	UPLC ‘Level 0’ data	Data	LONI	http://dx.doi.org/10.7303/syn7440355.1
ADMC Duke Biocrates P180 Kit Ultra Performance Liquid Chromatography Dictionary	Data dictionary for p180 UPLC	Data Dict	LONI	http://dx.doi.org/10.7303/syn7477316.1
P180FIALODvalues.csv	QC data for lower limit of detection	Data	AMPAD Knowledge Portal / Synapse	http://dx.doi.org/10.7303/syn9775685.1
P180UPLCLODvalues.csv	QC data for lower limit of detection	Data	AMPAD Knowledge Portal / Synapse	http://dx.doi.org/10.7303/syn9775688.1
ADMC Duke Biocrates P180 Kit Ultra Performance Liquid Chromatography Methods	Methods description for p180	Methods	LONI	http://dx.doi.org/10.7303/syn7477319.1
*Supplemental Metabolomics Files*				
ADMC About the Metabolomics Data	High level information about AD Metabolomics Consortium Data	Methods	AMPAD Knowledge Portal / Synapse	https://www.synapse.org/#!Synapse:syn8532154
ADMC_supplement.zip	Original MetIDQ software output files in.xlsx format, NIST and QC output from MetIDQ	Supplementary Data	LONI	On LONI under ‘ADMC Supplementary Materials’
ADMCDUKEP180FIA.LEVEL5.csv	Post-processed ‘Level 5’ file FIA	Data	LONI	http://dx.doi.org/10.7303/syn7440356.1
ADMCDUKEP180UPLC.LEVEL5.csv	Post-processed ‘Level 5’ file UPLC	Data	LONI	http://dx.doi.org/10.7303/syn7440357.1
ADNI_P180_LEVEL0_to_LEVEL1.R etc.	Data processing R scripts	Scripts	AMPAD Knowledge Portal / Synapse	http://dx.doi.org/10.7303/syn7354353
*Clinical and Medication files*				
ADNI_All_Clinical_Data_16May2016.csv	Clinical variables (a subset of ADNI's complete list) snapshot from May, 2016	Data	LONI	http://dx.doi.org/10.7303/syn7477271.1
Fasting Status.txt	Fasting status of participants at time of blood draw	Data	AMPAD Knowledge Portal / Synapse	http://dx.doi.org/10.7303/syn9774830.1
ADNI Key Clinical Variables Subset Data Dictionary.xlsx	Data dictionary for a key subset of variables in ADNI_All_Clinical_Data_16May2016.csv (for full version see ‘Data Dictionary [ADNI1,GO,2] (DATADIC.csv)’ on LONI)	Data dict	AMPAD Knowledge Portal / Synapse	http://dx.doi.org/10.7303/syn9758900.1
RECCMEDS.csv	Original medication data- all cohorts, all timepoints. NOT versioned.	Data	LONI	http://dx.doi.org/10.7303/syn7829508.1
Medication mapping pipeline files	Scripts and config files for medication concept mapping and classification	Scripts	AMPAD Knowledge Portal / Synapse	http://dx.doi.org/10.7303/syn7477310
ADMCADNI1SCPATIENTDRUGCLASSES.csv	Results file mapping participants to classes of drugs taken at baseline	Supplementary Data	LONI	http://dx.doi.org/10.7303/syn7440367.1
DOIs below point to objects in Synapse with direct links to files on LONI where applicable. All LONI data can also be accessed through http://adni.loni.usc.edu/data-samples/access-data/. Researchers may apply for data access at https://ida.loni.usc.edu/collaboration/access/appLicense.jsp.				

**Table 3:** Drug classes included in analysis, and the source terminologies in which they are defined

**CLASS NAME**	**SOURCE**
ACE inhibitors, plain	ATC
Adrenergic Uptake Inhibitors	MESH
Adrenergic and dopaminergic agents	ATC
Aldosterone antagonists	ATC
Alpha and beta blocking agents	ATC
Alpha glucosidase inhibitors	ATC
Alpha-adrenoreceptor antagonists	ATC
Aminoketone	DAILYMED,FDASPL
Angiotensin II antagonists, plain	ATC
Anti-Anxiety Agents	MESH
Anti-epileptic Agent	DAILYMED,FDASPL
Antiarrhythmics, class III	ATC
Antiarrhythmics, class Ib	ATC
Antiarrhythmics, class Ic	ATC
Anticholinesterases	ATC
Antidepressive Agents	MESH
Antidepressive Agents, Second-Generation	MESH
Antidepressive Agents, Tricyclic	MESH
Antihistamine	DAILYMED,FDASPL
Antipsychotic Agents	MESH
Atypical Antipsychotic	DAILYMED,FDASPL
Azaspirodecanedione derivatives	ATC
Barbiturates and derivatives	ATC
Benzodiazepine	DAILYMED,FDASPL
Benzodiazepine related drugs	ATC
Benzothiazepine derivatives	ATC
Beta blocking agents, non-selective	ATC
Beta blocking agents, selective	ATC
Biguanides	ATC,DAILYMED,FDASPL
Bile acid sequestrants	ATC
Butyrophenone derivatives	ATC
Carboxamide derivatives	ATC
Central Nervous System Depressants	MESH
Central Nervous System Stimulant	DAILYMED,FDASPL
Centrally acting sympathomimetics	ATC
Corticosteroids	ATC
Diazepines, oxazepines, thiazepines and oxepines	ATC
Digitalis glycosides	ATC
Dihydropyridine derivatives	ATC
Dipeptidyl peptidase 4 (DPP-4) inhibitors	ATC
Diphenylmethane derivatives	ATC
Ergot alkaloids	ATC
Fibrates	ATC
Fish Oils	NDFRT
HMG CoA reductase inhibitors	ATC
Heparins or heparinoids for topical use	ATC
Hydantoin derivatives	ATC
Imidazoline receptor agonists	ATC
Insulin	DAILYMED,FDASPL,NDFRT
Lithium	ATC,NDFRT
Local anesthetics	ATC
Melatonin receptor agonists	ATC
Mood Stabilizer	DAILYMED,FDASPL
Muscle relaxants	ATC
Nicotinic acid and derivatives	ATC
Nonsteroidal Anti-inflammatory Drug	DAILYMED,FDASPL
Norepinephrine Reuptake Inhibitor	DAILYMED,FDASPL
Organic nitrates	ATC
Other anti-dementia drugs	ATC
Other antidepressants	ATC
Other antipsychotics	ATC
Other blood glucose lowering drugs, excl. insulins	ATC
Other cardiac preparations	ATC
Other lipid modifying agents	ATC
Other potassium-sparing agents	ATC
Other psychostimulants and nootropics	ATC
Phenothiazines with piperazine structure	ATC
Phenylalkylamine derivatives	ATC
Purine derivatives	ATC
Pyrimidine derivatives	ATC
Renin-inhibitors	ATC
Serotonin Reuptake Inhibitor	DAILYMED,FDASPL
Serotonin and Norepinephrine Reuptake Inhibitor	DAILYMED,FDASPL
Sulfonamides, plain	ATC
Sulfonylureas	ATC
Sympathomimetic-like Agent	DAILYMED,FDASPL
Thiazides, plain	ATC
Thiazolidinediones	ATC,DAILYMED,FDASPL
Thyroid Hormone Receptor Agonists	NDFRT
Thyroid Hormone Synthesis Inhibitor	DAILYMED,FDASPL
Typical Antipsychotic	DAILYMED,FDASPL
Tyrosine	NDFRT
Vasodilator Agents	MESH
l-Thyroxine	DAILYMED,FDASPL
l-Triiodothyronine	DAILYMED,FDASPL
