# Supplementary material for: Targeted metabolomics and medication classification data from participants in the ADNI1 cohort
Source: Sci Data. 2017 Oct 17;4:170140. doi: 10.1038/sdata.2017.140 (PMC5644370; doi:10.1038/sdata.2017.140)
Supplement: Supplementary Table 1 [file sdata2017140-s2.docx]

**Supplemental Table 1**. Quality Control Pipeline Results for the 121 FIA-MS/MS Analytes from the p180 kit which pass the <40% missing values (LOD) statistical cut. “Analyte” represents the name of the metabolite, as defined in the Data Dictionary. “<LOD (%)” refers to the percentage of the samples which were found to be below the limit of detection (and thus replaced with a value of LOD/2) during the processing pipeline. “CV (%)” represents the average percent coefficient of variation for the blinded replicate samples. “ICC” represents the calculated intraclass correlation coefficient calculated between run 1 and run 2 of the blinded replicate samples.

| Analyte | < LOD (%) | CV (%) | ICC |
| --- | --- | --- | --- |
| C0 | 0 | 7.4 | 0.89 |
| C10 | 2.5 | 6.3 | 0.96 |
| C10.2 | 4.8 | 8.1 | 0.81 |
| C12 | 11.2 | 6.5 | 0.86 |
| C14.1 | 0 | 7.3 | 0.77 |
| C14.1.OH | 11.4 | 6.4 | 0.85 |
| C14.2 | 4.5 | 7.5 | 0.83 |
| C16 | 0.1 | 7.2 | 0.87 |
| C16.1 | 0.5 | 6.0 | 0.89 |
| C16.OH | 21.5 | 12.3 | 0.5 |
| C18 | 3.1 | 5.7 | 0.91 |
| C18.1 | 0.1 | 6.6 | 0.91 |
| C18.2 | 0 | 4.5 | 0.95 |
| C2 | 0 | 8.9 | 0.84 |
| C3 | 0.1 | 7.5 | 0.94 |
| C3.DC..C4.OH. | 0.4 | 12.4 | 0.84 |
| C4 | 0.1 | 8.1 | 0.92 |
| C5 | 19.3 | 7.4 | 0.93 |
| C5.1.DC | 26.3 | 14.8 | 0.49 |
| C5.DC..C6.OH. | 0.4 | 12.9 | 0.8 |
| C6..C4.1.DC. | 8.5 | 6.8 | 0.98 |
| C7.DC | 1.2 | 8.3 | 0.89 |
| C8 | 22.6 | 7.8 | 0.94 |
| C9 | 2.7 | 8.7 | 0.84 |
| lysoPC.a.C16.0 | 0.1 | 11.1 | 0.68 |
| lysoPC.a.C16.1 | 0.1 | 11.2 | 0.73 |
| lysoPC.a.C17.0 | 0.1 | 11.3 | 0.82 |
| lysoPC.a.C18.0 | 0.1 | 11.1 | 0.79 |
| lysoPC.a.C18.1 | 0 | 10.6 | 0.8 |
| lysoPC.a.C18.2 | 0 | 9.6 | 0.87 |
| lysoPC.a.C20.3 | 0.1 | 10.5 | 0.89 |
| lysoPC.a.C20.4 | 0 | 10.2 | 0.81 |
| lysoPC.a.C24.0 | 0.5 | 11.8 | 0.82 |
| lysoPC.a.C26.0 | 13 | 16.6 | 0.72 |
| lysoPC.a.C26.1 | 0.1 | 19.7 | 0.45 |
| lysoPC.a.C28.0 | 0.9 | 12.4 | 0.7 |
| lysoPC.a.C28.1 | 0.1 | 11.5 | 0.8 |
| PC.aa.C24.0 | 7.3 | 14.2 | 0.44 |
| PC.aa.C28.1 | 0.1 | 6.0 | 0.92 |
| PC.aa.C30.0 | 0.1 | 6.4 | 0.96 |
| PC.aa.C32.0 | 0.1 | 6.6 | 0.9 |
| PC.aa.C32.1 | 0 | 7.4 | 0.96 |
| PC.aa.C32.3 | 0.1 | 7.1 | 0.94 |
| PC.aa.C34.1 | 0 | 11.3 | 0.23 |
| PC.aa.C34.2 | 0 | 11.8 | 0.15 |
| PC.aa.C34.3 | 0 | 6.5 | 0.92 |
| PC.aa.C34.4 | 0 | 7.5 | 0.95 |
| PC.aa.C36.0 | 4.9 | 14.1 | 0.91 |
| PC.aa.C36.1 | 0 | 8.5 | 0.87 |
| PC.aa.C36.2 | 0 | 10.3 | 0.3 |
| PC.aa.C36.3 | 0 | 7.4 | 0.67 |
| PC.aa.C36.4 | 0 | 9.1 | 0.51 |
| PC.aa.C36.5 | 0 | 7.9 | 0.98 |
| PC.aa.C36.6 | 0 | 8.0 | 0.96 |
| PC.aa.C38.0 | 0.1 | 6.8 | 0.93 |
| PC.aa.C38.3 | 0 | 7.4 | 0.91 |
| PC.aa.C38.4 | 0 | 6.9 | 0.83 |
| PC.aa.C38.5 | 0 | 7.8 | 0.89 |
| PC.aa.C38.6 | 0 | 8.4 | 0.91 |
| PC.aa.C40.2 | 0.1 | 11.5 | 0.71 |
| PC.aa.C40.3 | 0.1 | 9.0 | 0.75 |
| PC.aa.C40.4 | 0 | 7.1 | 0.91 |
| PC.aa.C40.5 | 0 | 8.1 | 0.9 |
| PC.aa.C40.6 | 0.1 | 7.5 | 0.97 |
| PC.aa.C42.0 | 0.1 | 7.7 | 0.9 |
| PC.aa.C42.1 | 0.1 | 6.8 | 0.92 |
| PC.aa.C42.2 | 0.1 | 7.4 | 0.93 |
| PC.aa.C42.4 | 0.3 | 9.8 | 0.79 |
| PC.aa.C42.5 | 0.1 | 7.7 | 0.91 |
| PC.aa.C42.6 | 0.1 | 7.1 | 0.95 |
| PC.ae.C30.0 | 0.1 | 5.8 | 0.96 |
| PC.ae.C30.1 | 9.4 | 39.0 | 0.41 |
| PC.ae.C30.2 | 0.1 | 8.5 | 0.88 |
| PC.ae.C32.1 | 0.1 | 7.0 | 0.88 |
| PC.ae.C32.2 | 0.1 | 7.3 | 0.87 |
| PC.ae.C34.0 | 0.1 | 6.6 | 0.94 |
| PC.ae.C34.1 | 0 | 7.5 | 0.83 |
| PC.ae.C34.2 | 0 | 7.4 | 0.9 |
| PC.ae.C34.3 | 0 | 7.3 | 0.82 |
| PC.ae.C36.0 | 0.5 | 6.7 | 0.91 |
| PC.ae.C36.1 | 0.1 | 7.2 | 0.86 |
| PC.ae.C36.2 | 0 | 6.6 | 0.92 |
| PC.ae.C36.3 | 0 | 7.1 | 0.9 |
| PC.ae.C36.4 | 0 | 7.3 | 0.84 |
| PC.ae.C36.5 | 0 | 7.9 | 0.79 |
| PC.ae.C38.0 | 0.1 | 6.7 | 0.96 |
| PC.ae.C38.1 | 32.6 | 49.0 | 0.38 |
| PC.ae.C38.2 | 0.1 | 10.4 | 0.69 |
| PC.ae.C38.3 | 0 | 7.7 | 0.91 |
| PC.ae.C38.4 | 0 | 7.5 | 0.78 |
| PC.ae.C38.5 | 0 | 7.5 | 0.76 |
| PC.ae.C38.6 | 0 | 7.6 | 0.88 |
| PC.ae.C40.1 | 0.1 | 7.9 | 0.91 |
| PC.ae.C40.2 | 0 | 7.4 | 0.89 |
| PC.ae.C40.3 | 0.1 | 7.3 | 0.85 |
| PC.ae.C40.4 | 0.1 | 7.6 | 0.76 |
| PC.ae.C40.5 | 0 | 7.5 | 0.75 |
| PC.ae.C40.6 | 0 | 7.0 | 0.94 |
| PC.ae.C42.1 | 0.1 | 7.5 | 0.78 |
| PC.ae.C42.2 | 0.1 | 8.6 | 0.91 |
| PC.ae.C42.3 | 0.1 | 7.5 | 0.91 |
| PC.ae.C42.4 | 0.1 | 8.4 | 0.82 |
| PC.ae.C42.5 | 0.3 | 6.9 | 0.82 |
| PC.ae.C44.3 | 0.1 | 8.6 | 0.84 |
| PC.ae.C44.4 | 0.1 | 6.8 | 0.92 |
| PC.ae.C44.5 | 0.1 | 7.7 | 0.9 |
| PC.ae.C44.6 | 0.1 | 7.6 | 0.88 |
| SM..OH..C14.1 | 0.1 | 7.0 | 0.93 |
| SM..OH..C16.1 | 0 | 7.4 | 0.9 |
| SM..OH..C22.1 | 0 | 8.2 | 0.88 |
| SM..OH..C22.2 | 0 | 8.1 | 0.89 |
| SM..OH..C24.1 | 0.1 | 8.4 | 0.81 |
| SM.C16.0 | 0 | 7.2 | 0.85 |
| SM.C16.1 | 0 | 7.2 | 0.9 |
| SM.C18.0 | 0 | 7.6 | 0.88 |
| SM.C18.1 | 0 | 7.5 | 0.91 |
| SM.C20.2 | 0.1 | 8.0 | 0.93 |
| SM.C24.0 | 0 | 8.2 | 0.82 |
| SM.C24.1 | 0 | 8.7 | 0.77 |
| SM.C26.0 | 0.3 | 10.1 | 0.7 |
| SM.C26.1 | 0.3 | 8.8 | 0.86 |
