# Supplementary material for: Targeted metabolomics and medication classification data from participants in the ADNI1 cohort
Source: Sci Data. 2017 Oct 17;4:170140. doi: 10.1038/sdata.2017.140 (PMC5644370; doi:10.1038/sdata.2017.140)
Supplement: Supplementary Table 2 [file sdata2017140-s3.docx]

**Supplementary Table 2**. Quality Control Pipeline Results for the 32 LC-MS/MS Analytes from the p180 kit which pass the <40% missing values (LOD) statistical cut. “Analyte” represents the name of the metabolite, as defined in the Data Dictionary. “<LOD (%)” refers to the percentage of the samples which were found to be below the limit of detection (and thus replaced with a value of LOD/2) during the processing pipeline. “CV (%)” represents the average percent coefficient of variation for the blinded replicate samples. “ICC” represents the calculated intraclass correlation coefficient calculated between run 1 and run 2 of the blinded replicate samples.

| Analyte | < LOD (%) | CV (%) | ICC |
| --- | --- | --- | --- |
| ADMA | 0.4 | 12.9 | 0.51 |
| Ala | 0 | 5.3 | 0.84 |
| alpha.AAA | 18.9 | 7.2 | 0.53 |
| Arg | 0 | 5.3 | 0.92 |
| Asn | 0 | 4.9 | 0.87 |
| Asp | 0.1 | 10.4 | 0.86 |
| Cit | 0 | 7.5 | 0.94 |
| Creatinine | 0 | 4.1 | 0.98 |
| Gln | 0 | 5.1 | 0.74 |
| Glu | 0 | 9.0 | 0.81 |
| Gly | 0 | 6.2 | 0.97 |
| His | 0 | 5.7 | 0.75 |
| Histamine | 17.2 | 5.4 | 0.09 |
| Ile | 0 | 6.4 | 0.88 |
| Kynurenine | 0 | 8.4 | 0.93 |
| Lys | 0 | 6.6 | 0.77 |
| Met | 0 | 8.1 | 0.65 |
| Orn | 0 | 5.8 | 0.89 |
| Phe | 0 | 6.7 | 0.96 |
| Pro | 0 | 6.0 | 0.96 |
| Putrescine | 12 | 30.3 | 0.67 |
| Sarcosine | 0 | 12.1 | 0.85 |
| SDMA | 0 | 6.5 | 0.9 |
| Ser | 0 | 6.6 | 0.94 |
| Serotonin | 1.7 | 12.8 | 0.97 |
| Spermidine | 0.1 | 6.2 | 0.93 |
| t4.OH.Pro | 0 | 5.7 | 0.96 |
| Taurine | 0 | 4.0 | 0.96 |
| Thr | 0 | 4.3 | 0.9 |
| Trp | 0 | 6.6 | 0.9 |
| Tyr | 0 | 6.0 | 0.92 |
| Val | 0 | 6.6 | 0.84 |
